# A Surrogate Model Based on a Finite Element Model of Abdomen for Real-Time Visualisation of Tissue Stress during Physical Examination Training

**DOI:** 10.3390/bioengineering9110687

**Published:** 2022-11-14

**Authors:** Florence Leong, Chow Yin Lai, Siamak Farajzadeh Khosroshahi, Liang He, Simon de Lusignan, Thrishantha Nanayakkara, Mazdak Ghajari

**Affiliations:** 1 Dyson School of Design Engineering, Imperial College London, London SW7 2DB, UK; 2 Department of Electronic and Electrical Engineering, University College London, London WC1E 7JE, UK; 3 School of Engineering and Materials Science, Queen Mary University of London, London E1 4NS, UK; 4 Oxford Robotics Institute, University of Oxford, Oxford OX1 3PJ, UK; 5 Nuffield Department of Primary Care Health Sciences, University of Oxford, Oxford OX2 6GG, UK

**Keywords:** abdominal tissue simulator, medical training, finite element modelling, human–machine interaction, computational modelling

## Abstract

Robotic patients show great potential for helping to improve medical palpation training, as they can provide feedback that cannot be obtained in a real patient. They provide information about internal organ deformation that can significantly enhance palpation training by giving medical trainees visual insight based on the pressure they apply for palpation. This can be achieved by using computational models of abdomen mechanics. However, such models are computationally expensive, and thus unable to provide real-time predictions. In this work, we proposed an innovative surrogate model of abdomen mechanics by using machine learning (ML) and finite element (FE) modelling to virtually render internal tissue deformation in real time. We first developed a new high-fidelity FE model of the abdomen mechanics from computerized tomography (CT) images. We performed palpation simulations to produce a large database of stress distribution on the liver edge, an area of interest in most examinations. We then used artificial neural networks (ANNs) to develop the surrogate model and demonstrated its application in an experimental palpation platform. Our FE simulations took 1.5 h to predict stress distribution for each palpation while this only took a fraction of a second for the surrogate model. Our results show that our artificial neural network (ANN) surrogate has an accuracy of 92.6%. We also showed that the surrogate model is able to use the experimental input of palpation location and force to provide real-time projections onto the robotics platform. This enhanced robotics platform has the potential to be used as a training simulator for trainees to hone their palpation skills.

## 1. Introduction

General practitioners (GPs) often perform physical abdominal examinations on patients to identify physical signs that test or confirm diagnostic possibilities that have arisen from their patient’s history. Abdominal palpation is one of the most widely used examination procedures, which is difficult to learn as it involves multiple feedback modalities and sensory coordination, i.e., touch, hearing, and vision. Hence, the technique requires years of experience for practitioners to sharpen their skills and sensitivity for a reliable diagnosis. This is currently obtained through repetitive training on patients with real diagnoses, who give instant feedback about any discomfort through verbal communication [1,2], facial expression [3,4], and physical reactions such as muscle guarding [5,6]. However, repetitive practice on real patients is very challenging as patients have few stays of short duration in the hospital.

An elegant alternative to practicing on patients is simulation-based education (SBE) [7], whereby a proto-professional experience can be provided via utilizing manikin simulators [8] or virtual reality (VR) platforms [9]. The fabrication of internal organs is the key contribution of a physical manikin simulator. Multiple diseased liver models made of silicone can be designed as replacement units in a human-like physical abdominal simulator [10]. Soft robotic livers with tunable stiffness tumors made of granular jamming nodules have also been proposed to improve the controllability of the organ with more diverse pathological conditions rendered [8].

Alternatively, VR training systems allow higher flexibility in the simulated conditions, although the level of interactions is limited. Burdea et al. [11] implemented a haptic device with visual supplements on a computer screen to establish a virtual environment for digital rectal examination training. Haptic devices that render single-point interaction with six degrees-of-freedom actuators have been widely used to reassemble the input palpation force and location to output tissue compliance in VR-based training [12,13]. However, simulating the haptic sensing of touch based on VR images can be complex for palpation training, as it takes years of experience for trainees and clinicians to grasp accurate haptic sensation for diagnosis and decision-making [14].

Combining VR or augmented reality (AR) with a physical simulator provides advantages in gaining high-level interaction in a physical simulator and flexible feature rendering in virtual simulation as additional information for more effective training [15,16,17]. By incorporating visual information into physical training, medical trainees benefit from being able to associate haptic perception with the stress levels at an organ level. Such internal models mapping haptic perception to visual representations of mechanical stress during palpation will further enhance the ability to discriminate among different physiological events, such as the edge of the liver passing under the fingers during a breathing cycle, touching a swollen area of the intestine, etc.

This visual information will also provide trainees immediate insight on how the palpation would affect the internal abdominal tissue (e.g., too much pressure may cause discomfort to patients or impact on already damaged tissues), besides being an added potential for experienced clinicians to calibrate their examination technique. This is particularly advantageous in scenarios in which trainees are yet to be familiarised with the different multi-sensory perceptions, e.g., body language, facial expressions, and verbal signals shown by patients, from palpating on patients with actual conditions. However, currently there is no abdominal simulator for physical examination training that has the capability to provide such visual information to trainees especially in real time.

Computational modelling can be used to produce this important layer of information for the simulator. By using finite element modelling (FEM), we can predict tissue deformation under mechanical loading, such as palpation. Several FE human body models have been developed. For instance, a complete human body model named the total human model for safety (THUMS) model [18] by Toyota Motor Corporation was developed and validated for injury reconstruction and crash analyses, but it lacks details of the abdominal tissues. The Global Human Body Model Consortium (GHBMC) [19] has also developed a full human body model for impact studies in road traffic accidents. However, this model has a complex description of abdominal tissues and has been developed for high rate loading; hence, it is not suitable for palpation simulation. Therefore, we constructed a new model from CT scans of the human abdomen, allowing us to model different abdominal tissues for different physiological conditions, such as different stages of the respiratory cycle. Performing FE simulations for palpation requires a long run time and high computation, which is not desirable especially when real-time information is needed for immediate training feedback.

Surrogate models based on machine learning (ML) methods can potentially help us build accurate and fast models to integrate with a physical setup for real-time palpation training. Machine learning has been used previously to build fast and accurate surrogate models by using data obtained from FEM simulations. An artificial neural network (ANN) was trained in [20] to estimate stress distribution in the aorta, and in [21,22] to simulate deformation in soft tissue in real time. Other studies implemented a convolution neural network (CNN) technique for fast visualisation of soft tissue behaviour from FEM simulations, such as liver deformation [23,24]. These previous studies have developed surrogate models for real-time surgical planning, computer-guided surgery, and guided tumour irradiation. However, there are currently no surrogate models available for palpation.

**Figure 1:**
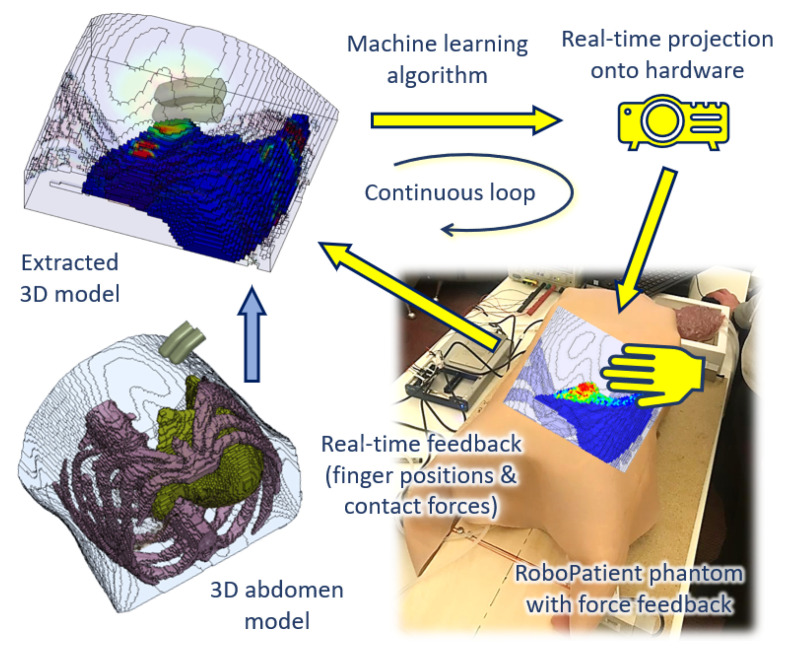
An overview of the real-time internal abdomen visualisation with respect to force feedback from the robotic patient simulator based on the surrogate model. The surrogate model is trained by using a machine learning algorithm to render tissue deformation by using the input force and palpation location performed by the user.

In this paper, we proposed a new ANN-trained surrogate model based on FEM simulations as shown in Figure 1. The FE simulations were performed by using a new model of the abdomen developed from CT scans. We tested whether the surrogate model could accurately predict the stress distribution at the edge of the liver, which is a key area in a physical examination. We also showed that the surrogate model could provide real-time predictions. Finally, we demonstrated that the surrogate model could be integrated with a physical palpation platform to provide visual information on tissue deformation.

## 2. Methodology

The development of the RoboPatient simulator involves the integration between the hardware platform with force feedback, and the software, i.e., the construction of a surrogate model based on FEM simulations of abdominal palpation. These stages are described in the following subsections.

### 2.1. Finite Element Model Setup

**Figure 2:**
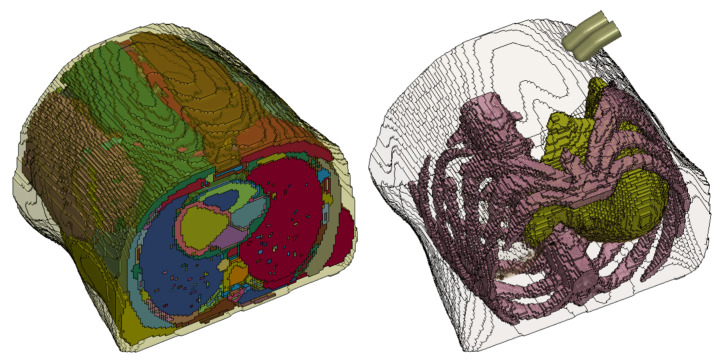
Abdomen model. (**a**) High-fidelity model generated by the XCAT program. (**b**) Simplified abdomen model with lumped abdominal tissue, ribcage, liver, and dummy fingers.

An anatomically detailed 3D model of the abdomen was extracted from computerized tomography (CT) scans of the human abdomen by using 4D Extended Cardiac-Torso (XCAT) Phantom software [25] configured to capture the scan images with respiratory motion at full exhalation. The generated scan sequences were then post-processed in ImageJ software to produce 32-bit real image sequences at a resolution of 141×141 pixels to be converted into a 3D human abdomen model. This full abdomen model was then segmented and meshed (Figure 2a) for FEM simulations.

We used an image-based meshing algorithm as described in Ghajari et. al. [26] to develop the FE model. In this method, the coordinates of each voxel of the image was used to generate a hexahedral FE. Different anatomical regions of the image were assigned different part numbers in the mesh file. This meshing process led to jagged edges on the surface of the model. The mesh-smoothing algorithm, explained in Ghajari et. al. [26] was used to smooth the mesh. The model was then simplified by grouping all internal organs, except the ribs and liver, into a "lumped" abdominal model (see Figure 2b) with the focus on liver deformation during palpation in this study. This was done to reduce the complexity of materials and contact interactions among the many organs within the abdomen in the model.

The simulations were set up in LS-Prepost (Livermore Software Technology Corporation—LSTC, Livermore, CA, USA) by using material properties validated for the flesh (i.e., simplified rubber/foam) in [27,28,29] to represent the lumped abdominal tissue, and those for the ribs (i.e., piecewise linear plasticity) in [29,30,31] (see Table 1). The liver was modelled as a visco-hyperelastic material with parameters reported in [32,33]. The Ogden strain energy function in Equation (1) was used to model the hyperelastic part of the liver tissue response,

(1) $$\Psi^{\infty }=\sum_{p=1}^{n}\frac{\mu_{p}}{\alpha_{p}}\left(\lambda_{1}^{\alpha_{p}}+\lambda_{2}^{\alpha_{p}}+\lambda_{3}^{\alpha_{p}}-3\right),$$

where $\lambda_{i}$ are the principal stretches with i=1,2,3, and $\mu_{p}$ and $\alpha_{p}$ are the material constants, which we obtained by fitting the model to experimental stress/strain curve provided in [32]. Equation (2) incorporates the strain rate dependency of the tissue into the model,

(2) $$S_{\left(t\right)}=S^{\infty }+\int_{0}^{t}G\left(t-T\right)\frac{\partial E(T)}{\partial T}dT,$$

where $S^{\infty }$ is the long-term second Piola–Kirchhoff stress tensor, *E* is the Green–Lagrange strain tensor, and the relaxation function G(t) is represented by the Prony series given by Equation (3),

(3) $$G\left(t\right)=\sum_{i=1}^{n}G_{i}e\left(\frac{-t}{\tau_{i}}\right),$$

in which $G_{i}$ and $\tau_{i}$ are the shear moduli and the decay constant, respectively. The material constants for the visco-hyperelastic liver tissue are given in Table 2.

An FE model of two fingers were added to the abdomen model to simulate palpation. As the deformation of the finger is negligible compared with the soft abdominal tissue, they are modelled as a rigid part. The surface-to-surface penalty contact algorithm was used to define the contact between the fingers and abdomen.

### 2.2. Finite Element Simulations

FE simulations often require long run-time and high computation especially when the model is highly complex with respect to the number of parts, contact surfaces, and elements. We explored several ways to further simplify the abdomen model for simulations with the aim to reduce computational time.

**Figure 3:**
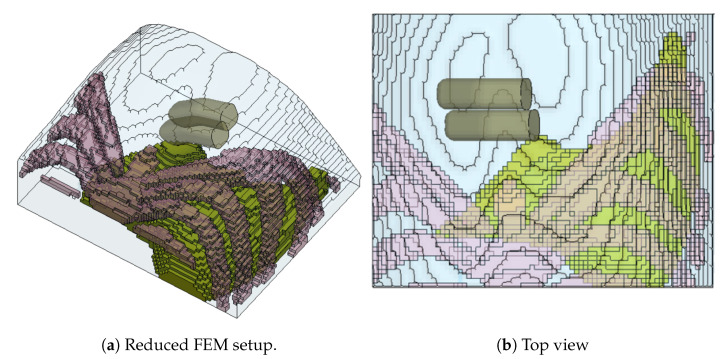
Simulation setup in LS Prepost. (**a**) Reduced version of the simplified abdomen model for simulations. (**b**) Top view of the abdomen model, focusing on the liver organ.

We cropped out a reduced model comprising the top half of the liver and the surrounding abdominal tissue (Figure 3), reducing the number of elements from 547,250 elements to 112,955 elements. We simulated 15 mm of finger indentation onto both the newly reduced model and the abdomen model depicted in Figure 2b to verify the difference in the behaviour of both the full simplified model and the reduced model. We found a negligible difference between the models in terms of the contact force (less than 0.12 N), but there was a significant reduction in the simulation time from about 18.5 h down to 7.5 h.

We also performed a simulation on the reduced model with indentation over a longer duration of 500 ms (simulation duration = 57 h) to observe the difference in the tissue response with the simulation at 25 ms. The effect of the palpation speed on the tissue response was also found to be negligible. It should be noted that we focused on the transient response during indentation, thus not taking into account tissue relaxation.

**Figure 4:**
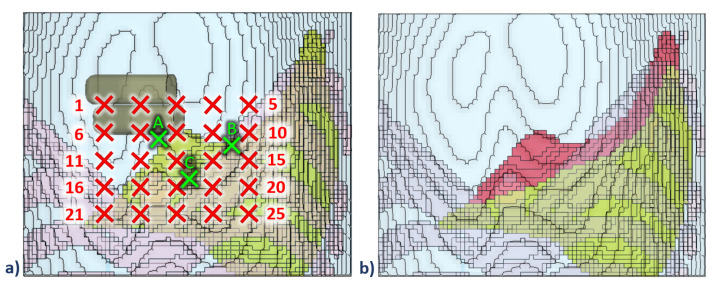
(**a**) Simulation sequence with fingers indentation up to 15 mm on locations across the liver region on top of the abdomen. The simulation started with the fingers at position 1 on the top-leftmost location, and ended with the fingers at position 25 on the bottom-rightmost location. The red crosses represent locations with FEM simulation data for ANN training, whereas the green crosses at locations A, B and C are taken as the data unseen by the ANN algorithm for testing. (**b**) The region of interest (ROI) on the surface edge of the liver tissue (depicted in red) with 377 elements for surrogate model training. The ribs are not shown as to not occlude the view of the liver.

The palpation simulations for our application were then performed with finger indentations up to 15 mm onto the abdominal locations above the liver region, depicted by the red crosses in Figure 4a. We developed a framework in Matlab to generate palpation FE models for each of these indentation locations. The Matlab code generates the FE models, runs FE simulations, and saves the results in text files. Each simulation took approximately 1.5 h on our high performance computing (HPC) unit with 20 cores and 24 gigabytes of memory. The final datasets generated by using this framework contained the stress/strain of every element of the region of interest (ROI), as shown in Figure 4, as a function of the indentation locations and the fingers contact forces for each simulation time stamp. The information in the datasets forms the basis of the surrogate model for the ANN training in the next step.

### 2.3. Construction of the Surrogate Model

**Figure 5:**
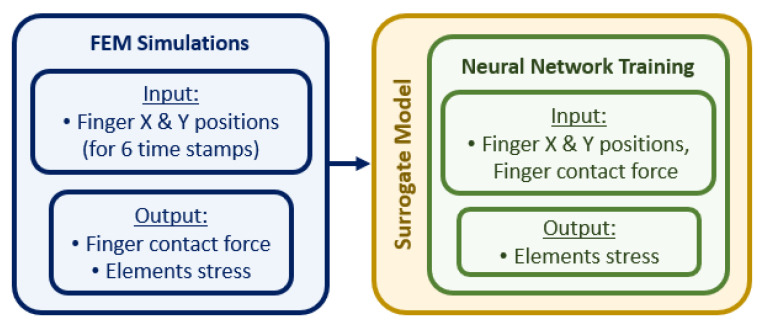
Flowchart on the use of information gathered from the FEM simulations to construct the surrogate model for effective ANN training.

The surrogate model links the input parameters in the FE simulations, i.e., the positions of the fingers in the x and y axes and the contact force on the tip of the fingers, to the output parameters of the simulation, i.e., the stress tensor of the elements in the liver, as shown in the flowchart in Figure 5. As our aim is to predict the stress distribution of the liver tissue at indentation locations that have not been simulated with the FE model, we trained an ANN described in the next section by using the information we obtained from our simulations.

We focused on the edge of the liver as the region of interest, which has 377 elements (see Figure 4b). The stress tensors of each solid element at each time step was used to compute the maximum principal stress (Equation (4)):

(4) $$\begin{array}{cc} \sigma_{ij} & = [\begin{array}{ccc} \sigma_{xx} & \sigma_{xy} & \sigma_{xz}\\ \sigma_{yx} & \sigma_{yy} & \sigma_{yz}\\ \sigma_{zx} & \sigma_{zy} & \sigma_{zz} \end{array}]\\ eig [\sigma_{ij}] & = [\begin{array}{ccc} \sigma_{1} & 0 & 0\\ 0 & \sigma_{2} & 0\\ 0 & 0 & \sigma_{3} \end{array}] \end{array},$$

where $\sigma_{ij}$ is the stress tensor, and its eigenvalue gives a diagonal matrix consisting of $\sigma_{1}$, $\sigma_{2}$, and $\sigma_{3}$, which are the minimum, intermediate, and maximum principal stresses, respectively. In this study, we used the maximum principal stress, $\sigma_{3}$.

### 2.4. Surrogate Model Training

The surrogate model was trained with 150 datasets consisting of 25 palpation locations (depicted by red crosses in Figure 4a) at six time stamps (0 ms, 5 ms, 10 ms, 15 ms, 20 ms and 25 ms). The input to the model were location and force of indentation, and output was the stress in the liver elements. Data from three other palpation locations (depicted by green crosses in Figure 4a) were used to test the network.

**Figure 6:**
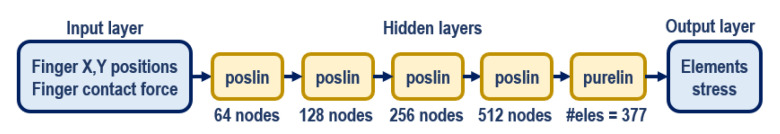
The ANN architecture for surrogate model training with fingers x and y positions and fingers contact force as input to the network, five hidden layers, and the maximum principal stress distribution on the elements as the output. The first four hidden layers with 64, 126, 256, and 512 nodes, respectively, implement positive linear (poslin) activation functions, and the final hidden layer which has 377 nodes corresponding to 337 elements in the ROI (#eles) uses the hard-limit linear function (purelin) for the network training.

An ANN with multi-layer perceptron was developed in Matlab for the surrogate training by using the ANN training toolbox. The network (Figure 6) has five hidden layers which use positive linear (poslin) activation functions, and the final hidden layer uses a hard-limit linear (purelin) function. The training implements gradient descent, specifically that with momentum and adaptive learning rate backpropagation algorithm. This network architecture was chosen based on the balance between the network complexity and its performance. The training process took approximately 16 min to complete with the datasets extracted from all the FEM simulation on a desktop computer (Intel(R) Xeon(R) CPU E5-2623 v4 @ 2.60 GHz—dual processors, 128 GB RAM). The trained network was then tested with the test dataset, and its the accuracy was evaluated.

### 2.5. Integration of the Surrogate Model and Force Sensing Platform

**Figure 7:**
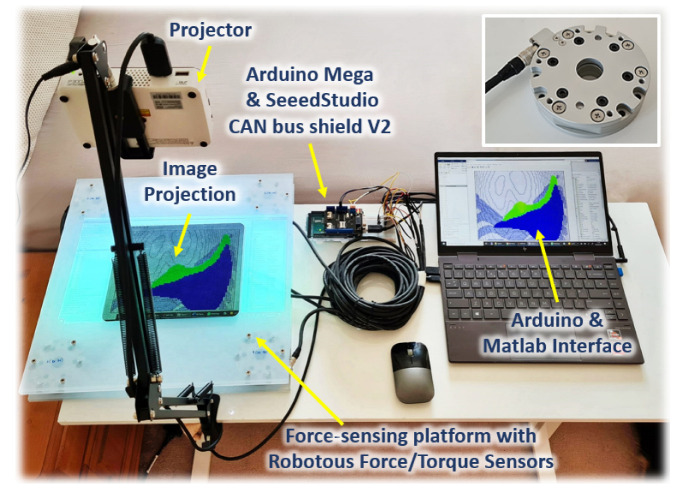
The overall hardware integration, with the 2D image of the surrogate model projected onto the force-sensing platform which is equipped with a Robotous 6-axis force/torque sensor (inset, top right corner) at each corner of the platform to obtain real-time force and position feedback.

The surrogate model was then integrated with a force-sensing platform. The overall experimental setup is shown in Figure 7. The force-sensing platform is constructed from perspex pieces of 400 mm × 400 mm × 10 mmin dimensions. The platform is equipped with four Robotous RA80-6A01 force-torque sensors, each attached to one corner of the platform. These sensors are connected together by CAN buses via SeeedStudio CAN Bus Shield V2 to Arduino Mega 2560 for measurement readings. The readings from each sensor are combined to provide force measurements when fingers are in contact with the platform.

Calibrations are also performed by using the readings of each sensor to determine the positions of the fingers during palpation. These position and force readings are obtained continuously in real time and are sent to the surrogate model to predict the stress/strain field at the edge of the liver. The stress distribution is mapped to a colour scheme and projected onto the platform for real time visualisation of the changes by using an AAXA P300 NEO Pico LED Video Projector.

## 3. Results and Discussion

### 3.1. Fe Predictions of Liver Stress Due to Palpation

**Figure 8:**
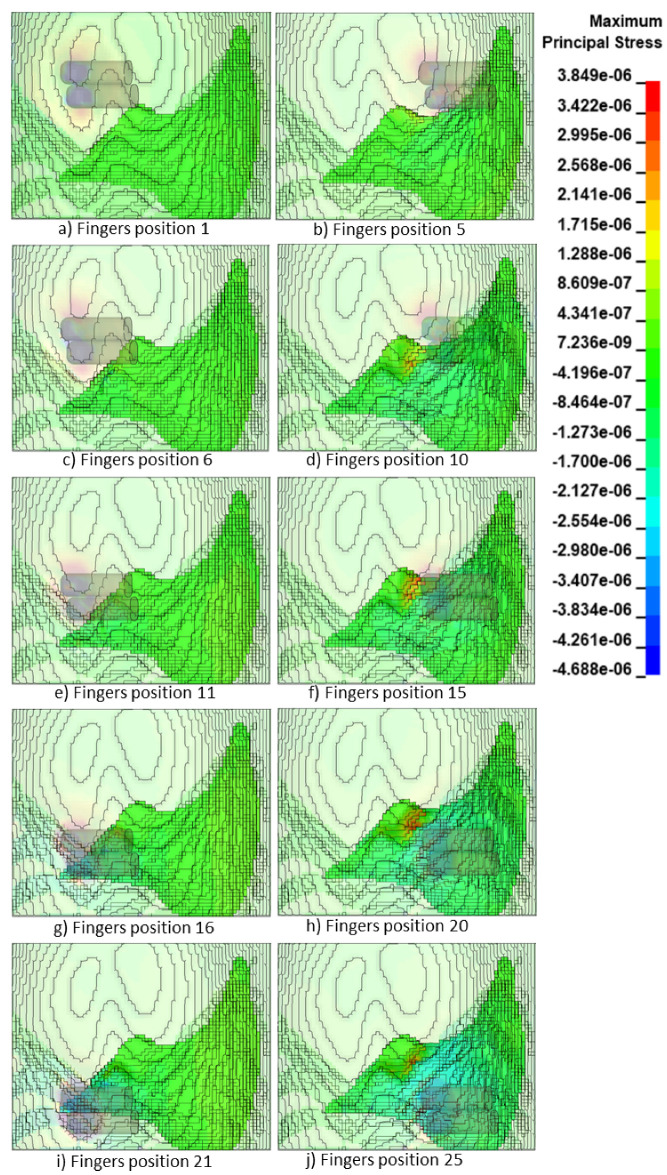
Simulation results illustrating the maximum principal stress distributions (in GPa) on the elements of the liver tissue at maximum indentation of 15 mm for each of the first and last horizontal positions of fingers according to Figure 4a.

Figure 8 shows the maximum principal stress distribution (in GPa) within the liver at the maximum indentation (15 mm) at the final simulation time stamp (25 ms). The simulation results here are shown for selected simulation cases, i.e., the first and last indentation locations in each horizontal line according to Figure 4a for illustration due to space constraint. The range of the maximum principal stress displayed in the legend covers the maximum and minimum values of the overall 25 sets of simulation data across all six time stamps, i.e., 3.8 KPa and −4.7 KPa, respectively. As expected, the maximum stress was predicted near the edge of the liver and when the fingers were passing across this region.

The new FEM of the human abdomen we constructed provides us with detailed insight of the internal tissue behaviour for palpation as opposed to the existing models which are established for specific purposes, such as high-impact analyses [18,19]. However, FEM simulations are highly time-consuming, at least 1.5 h for each palpation location, rendering them impractical for real-time predictions.

### 3.2. Surrogate Model Validation and Performance

**Figure 9:**
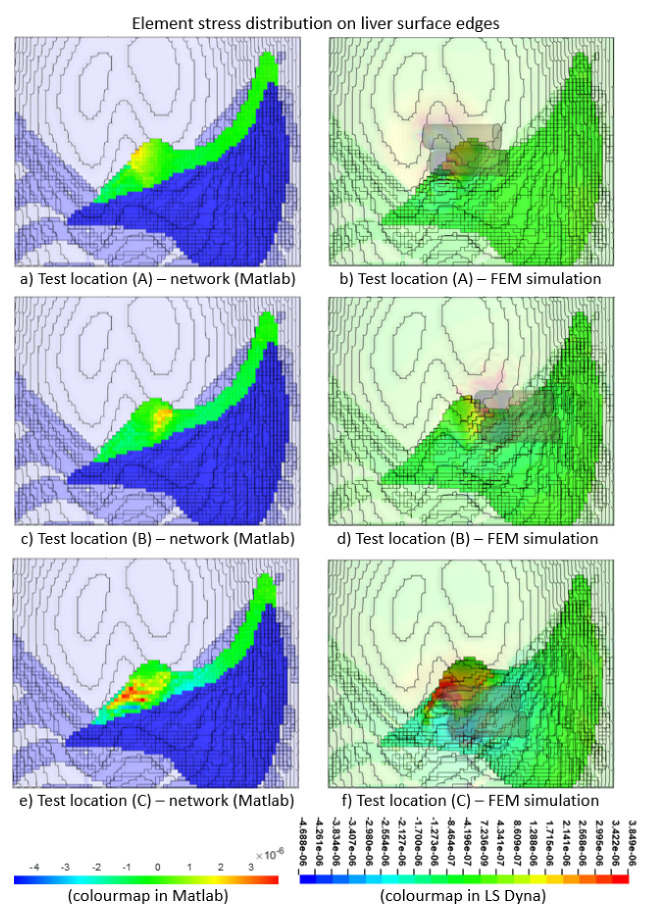
Validation of the ANN test output (**a**,**c**,**e**), focusing on the edge of the liver against FEM simulations (**b**,**d**,**f**) for cases A, B, and C, respectively, according to test locations shown in Figure 4a. (Note: The colour map illustrating the maximum principal stress (*GPa)* in the ANN is not exactly the same as that in LS-Dyna; hence, there is a difference between the colour distributions in the images generated).

**Figure 10:**
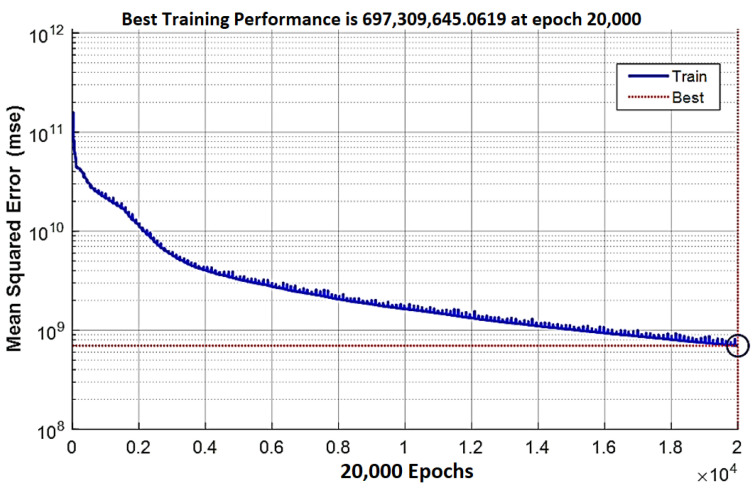
The mean squared error reduction history during the ANN training across all 377 elements in the region of interest.

We validated the performance of the surrogate model against the FEM simulations. The validation showed comparable stress distribution onto the liver edge across the ROI, with correct prediction of areas of high stresses (see Figure 9). The performance of the surrogate model was evaluated on the test dataset by using Equation (5),

(5) $$Fit=1-\sqrt{\Sigma {\left(y_{k}-{\hat{y}}_{k}\right)}^{2}/{\left(y_{k}-\bar{y}\right)}^{2}},$$

where *y*, $\hat{y}$ and $\bar{y}$ are the actual, estimated, and average stress values in testing stage respectively, and *k* is the element number in the FE model. The performance of the surrogate model was 92.64%. The training performance is shown in Figure 10, with the mean squared error of all 377 elements within the ROI approaching the best threshold at epoch 20,000.

The entire ANN training process on the surrogate model only took approximately 16 min, and the following predictions of the stress distributions across the ROI occur instantly with respect to the forces and positions of the fingers when in contact with the force-sensing platform, without having to execute FE simulations each time. This process provides us with the advantage of immediate visual feedback for palpation training.

### 3.3. Implementation on Force-Sensing Platform

**Figure 11:**
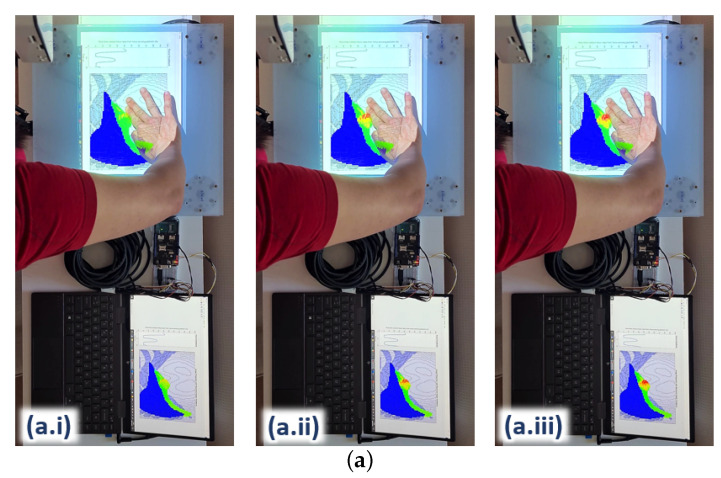

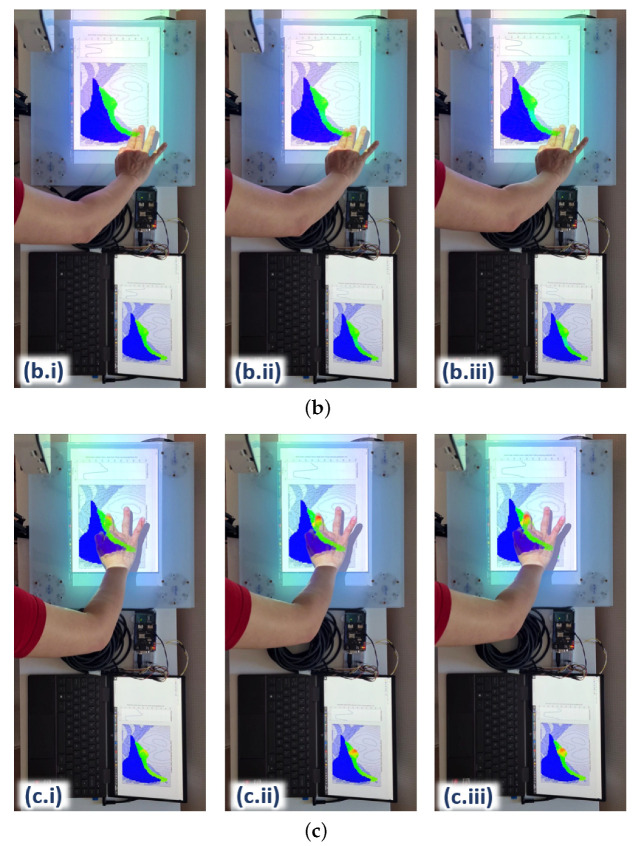
Palpation onto the force-sensing platform with real-time force feedback and stress distribution projection onto the platform at locations A, B, and C. (**a**) Palpation location A with approximate incremental forces (**a.i**) 6 N, (**a.ii**) 27 N, and (**a.iii**) 37 N. (**b**)Palpation location B with approximate incremental forces (**b.i**) 6 N, (**b.ii**) 27 N, and (**b.iii**) 37 N. (**c**) Palpation location C with approximate incremental forces (**c.i**) 6N, (**c.ii**) 27N, and (**c.iii**) 37N.

Upon the integration of the surrogate model with the hardware platform, the ANN takes in real-time contact force and x–y position readings from the force sensors incorporated on the platform (see Figure 7) as input. The changes in the internal element stress distribution within the ROI are then projected onto the hardware platform for real-time visualization during user training. Figure 11 shows three examples of the 2D rendering of the stress distribution from the trained ANN on the hardware platform during real-time force feedback. The image frames are taken at three different palpation locations, i.e., A, B and C, at three approximately similar contact forces, i.e., (i), (ii) and (iii), respectively, to observe the stress distribution on the liver edge.

The stress distribution on the liver tissue is highly dependent on the 3D model of the liver within the abdomen. For instance, some parts of the liver are closer to the surface of the abdomen and not covered by the ribcage (e.g., where palpation at location A is); hence, this part of the liver tissue is more responsive to palpation, visualising a higher stress distribution around the vicinity. The part of the liver tissue, where location B is, appears to be deeper in the 3D abdomen model; hence, it would need stronger palpation or higher force to reach the same stress distribution on the liver as it is with case C. In addition, the force would have been attenuated to a certain degree by the thick abdominal tissue above that part of the liver.

As for the liver region around location C, most of the liver tissue is located beneath the rib cage in the 3D model; hence, more force would also be needed to reach a higher stress distribution on the liver tissue. These differences in the stress distributions around the abdomen clearly demonstrate that the surrogate model trained by the ANN matches the behaviours of the FE model in the aspects of palpation locations as well as the 3D of the model (e.g., the tissue depth within the model).

The integration of the surrogate model with the force-sensing platform to provide real-time visual feedback provides us with several benefits. Our new abdomen FEM has the capability to generate high-fidelity tissue behaviours comparable to existing FE models [18,19]. In addition, our ANN-trained surrogate model based on the FEM enables us to augment information from the visual-contact force interaction as seen in high-level interventions such as surgical planning and computer-aided surgical procedure [22,23,24]. This is a promising application for palpation training so medical trainees and practitioners have an added visual aid to help regulate their palpation behaviours with respect to the impact onto the internal organs during palpation.

### 3.4. Limitations and Future Work

Currently, the RoboPatient simulator is tested only with the perspex force-sensing plate for feasibility study. With the surrogate model trained based on the FEM simulations results, the force and position feedback on the force-sensing platform are sufficient to reflect the stress distribution on the 2D image projection. An abdomen phantom, moulded with a liver phantom within (both can be made by casting EcoFlex silicone resin) to match the FE model in 2D or 3D configuration can be conveniently included for a more realistic feel. The phantom can also be customised to emulate various pathological conditions for diverse training experience, such as added tumorous tissues of different stiffness levels by using a modular granular jamming design [8].

The FE model of our abdomen was generated by using XCAT software from a single male human body based on the 3D Visible Human Project [34]; hence, it does not take into account physiological variations, such as the visceral fat in different human bodies. More investigation is required to include these variations into our FE model configurations to provide a more realistic abdominal model for a larger range of palpation training. In addition, the FE model was extracted from the CT scan at a single respiratory frame. Even though this model provides a sufficient representation of the abdominal tissue dynamics during palpation for our feasibility study, it would be more realistic to take into account the respiratory cycle in our simulations. Customised tissue abnormalities, such as tumours within the liver tissue with different severity can also be included into the FE models for more advanced training programmes. These features can then be added into the surrogate model and ANN training, as well as the abdomen phantom to produce a more authentic visualisation and palpation feedback.

When palpating onto the force-sensing platform, a slight latency in the range of 200–500 ms during the visual projections was observed, taking into account the duration of 150 ms our brains take to process visual information [35]. This slight latency does not affect the feedback of the force sensor input (i.e., contact force and fingers location) to the surrogate model and the tissue deformation rendering, but it could be improved for a smoother visual projection. This slight latency could be due to the communications within the hardware and software integration (i.e., Arduino, SeeedStudio CAN Bus Shield, Matlab) and the refresh rate in the projector. It can be improved by hardware enhancement (e.g., a better processor) and optimization of the algorithms. We can also consider designing a more modular ANN or a cluster of localised networks that is more region-oriented to provide faster response, and, hence, a faster training rate. This would be advantageous in allowing us to expand the ROI to the entire liver or abdomen.

A user study will be performed with the involvements of medical trainees and practitioners to assess the level of improvements in their palpation skills when given the added visual feedback. We are interested to observe the changes in their palpation skills, such as the reconditioning of fingers’ configuration and regulating palpation forces with respect to the real-time projection of internal stress distribution, as well as the enhancement in their perception when other feedback (e.g., haptic or facial expressions) are involved.

## 4. Conclusions

In this paper, we present a neural network-trained surrogate model for real-time visualisation of internal abdominal stress distribution during manual palpation of the abdomen. This surrogate model can be used to assist medical trainees to use a robotic patient phantom to associate their palpation forces with the internal organ deformation. Such visual feedback can be potentially used as a gender-ethnicity neutral intervention to train medical students to mitigate biases in other inputs such as facial or auditory expressions of pain during physical examination. It is beneficial for trainees who have not yet attained much experience involving multiple motor-sensory systems when palpating on actual patients, for instance visual and auditory signals (i.e., to appropriately understand facial and verbal expressions of patients) and the sense of touch (i.e., in feeling the different textures and stiffness of tissue as well as patient’s motions such as muscle contractions). Moreover, the added visual feedback on internal tissue can add potential for experienced clinicians to calibrate their examination techniques to improve their skills in diagnosis and decision-making. The benefits of this RoboPatient simulator with the added visual feedback will be evaluated through a user study involving medical trainees and practitioners.

A high-fidelity FE model of the abdomen was generated and used to predict tissue dynamics during palpation. We constructed a surrogate model based on FEM simulation results, and trained it with an ANN to produce rapid results with a performance fit accuracy of 92.64%. This enables accurate real-time estimation of the internal tissue stress given a real-time palpation force. The ability of the surrogate model, which represents the 3D high fidelity FE abdomen model to take in real-time physical palpation input from the hardware platform gives us the advantage of an augmented visuo-haptic feedback as compared to a pure virtual interaction. The slight latency during real-time projection can be improved by further optimising the code implementation, as well as the hardware integration.

The ultimate aim, which we are currently working toward, is to incorporate a layered abdominal tissue phantom, moulded with liver phantom with the force-sensing platform, for a more realistic feel during manual palpation as shown in Figure 1. To enhance our FE abdomen model, we are also working on incorporating respiratory cycles into the FEM simulation to provide a better understanding on tissue behaviour when the abdomen is being palpated in normal breathing conditions. Different tissue conditions, such as a liver with tumours of different stiffness and sizes at various locations, can also be added into the FEM simulations and the ANN surrogate model for a better sense of finger-tissue interaction, hence providing a more complete palpation training experience.

## Figures and Tables

**Table 1:** Material properties of the lumped abdominal tissue (flesh) and the ribs.

**Flesh**				
ine Mass	Bulk	Damping	Shear	
Density	Modulus	Coefficient	Modulus	
kg/mm^3^	(GPa)		(GPa)	
1.06 × 10^−6^	2	0.4	400 × 10^−5^	
**Ribs**				
Mass	Young’s	Poisson	Yield	Tangent
Density	Modulus	Ratio	Stress	Modulus
kg/mm^3^	(GPa)		(GPa)	
1.00 × 10^−6^	0.04	0.45	0.0018	0.001

**Table 2:** Material properties of the liver tissue.

Mass	Poisson				
Density	Ratio	$\mu_{p}$	$\alpha_{p}$	$G_{i}$	$\tau_{i}$
$\left(\mathrm{kg}/{\mathrm{mm}}^{3}\right)$		(GPa)		(GPa)	
$1.05\times 10^{-6}$	0.49	$8.914\times 10^{-8}$	1.0000	$6.97010\times 10^{-6}$	10
		$9.965\times 10^{-9}$	19.0656	$5.83270\times 10^{-5}$	$10^{2}$
		$-9.275\times 10^{-8}$	−10.9604	$3.52910\times 10^{-5}$	$10^{3}$

## Data Availability

The data presented in this study are available on request from the corresponding author.
